# Is playing violent video games a risk factor for aggressive behaviour? Adding narcissism, self-esteem and PEGI ratings to the debate

**DOI:** 10.3389/fpsyg.2023.1155807

**Published:** 2023-07-05

**Authors:** Szymon Zbigniew Olejarnik, Daniela Romano

**Affiliations:** ^1^Department of Psychology and Language Sciences, University College London, London, United Kingdom; ^2^Department of Information Studies, University College London, London, United Kingdom; ^3^Institute of Artificial Intelligence, De Montfort University, Leicester, United Kingdom

**Keywords:** aggressive behaviour, violent video games, narcissism, self-esteem, social psychology

## Abstract

**Introduction:**

Aggressive behaviour is a challenge for society. There continues to be considerable debate over whether the consumption of violent video games affects aggression, as violent video game content has become more accessible in recent years due to the growing use of online distribution platforms. Personality traits often linked to aggression, such as narcissism and self-esteem, have been considered in the context of violent video game play and their relationship with aggression.

**Methods:**

We surveyed an international population of 166 game players on their personality traits and their three favourite video game choices, which were classified as violent or non-violent, using Pan European Game Information (PEGI) 16 and 18 ratings.

**Results:**

We found that violent video game choice is a predictor of verbal aggression alongside narcissism, and hostility alongside self-esteem. A categorical regression highlighted the desire to impersonate society’s undesirable role models (e.g., ‘be a thief or a killer’) as one of the motivations for aggression and violent video game choice.

**Discussion:**

These findings show that video game violence should be considered a risk factor for aggression, as in other violent media, as it provides a social reinforcement of aggressive behaviour and observational learning of aggressive models, calling for the introduction of stricter online age verification procedures on online game platforms to safeguard children from violent video game content; and increased use of parental controls on content fruition. More granularity should be considered in the PEGI classifications.

## 1. Introduction

Aggressive behaviour has been most commonly defined in social psychology as any behaviour performed with the intention to harm another human being, who is motivated to avoid that harm (Bushman and Huesmann, 2010). Aggression can appear in many forms, “ranging from relatively minor acts (such as name calling or pushing) to more serious acts (such as hitting, kicking, or punching) to severe acts (such as stabbing, shooting, or killing)” (page 2, Sturmey et al., 2017). The general aggression model (GAM) (Allen et al., 2018) considers aggression as a compound of social, cognitive, personality, developmental and biological factors. It explains how appraisal and the decision process are influenced by the circumstances, as well as one’s cognition, feelings and arousal, which in turn influence aggressive or non-aggressive behavioural outcomes.

To date, experiments into the effects of video games on aggression have produced mixed results. In recent literature, a longitudinal study by Kühn et al. (2019) followed players of Grand Theft Auto V and The Sims 3 over the course of 2 months. Grand Theft Auto V is a vivid adventure game for a mature audience about the life of three criminals, a con artist, a drug lord, and a street hustler, described as containing intense violence, blood and strong language. The Sims 3 is a sandbox game where the players create and control the daily activities of a character’s life within a muti-agent world. The game allows swearing and violence in a manner that is more similar to cartoon humour. The researchers found no effect of either game on aggression when compared to the baseline. Also, an investigation into Korean adolescents found that violent gameplay lowered physical aggression in more dedicated players (Lee et al., 2020). Virtual reality technology is now available for video gameplay, providing a more immersive experience, thus inducing a possible increase in physiological responses and arousal. Ferguson et al. (2022) studied the effect of violent and non-violent virtual reality games. The games were a first-person shooter game Rush of Blood, with several levels, and a racing game Driveclub with several difficulty settings. They randomised the effect of both violence and frustration and found no appreciable impact on aggressive affect or behaviour.

Dowsett and Jackson (2019) argued that competitiveness increased aggression rather than violent video game content. However, Dickmeis and Roe (2019) found those video game genres classified as both violent and competitive (e.g., first-person shooters) were related to self-reported physical aggression, with both factors having influenced aggression. An investigation into Chinese adolescents found that moral disengagement was a significant mediator of longitudinal violent video game exposure and aggression, suggesting it was desensitisation to violent content that enabled heightened aggression in the context of video games (Teng et al., 2019). This provides an external factor of desensitisation that results in heightened aggression rather than violent video games themselves causing aggression. In addition, Agustarika and Adam (2020) found a significant effect between violent behaviour and online game addiction in Indonesian high school students.

Devilly et al. (2021) investigated the effect of violent and non-violent video games on anger and behavioural aggression. They argued that the GAM considers personological factors, such as personality and behaviour, only a minor component in the aggression model, which focuses on the learning afforded with each new exposure to violent media. They found that anger-inducing video games influenced mood and that both behavioural impulsivity and frustration with media increased anger, while there was no correlation between video game usage and behavioural aggression. They concluded that personality and frustration were predictors of anger and aggression.

All literature findings above present a convoluted picture that still does not allow us to answer whether violent video games cause aggression easily. Previously, Elson and Ferguson (2014) sought to summarise the results of experiments on the relationship between video games and aggression over a 25-year period. The overarching conclusion was that there was insufficient evidence to make solid conclusions on this subject, suggesting that the field must engage in dialogue to uncover the real cause of the matter without making video games into a moral panic, as is often the case in the media. Indeed, they noted a court case concerning selling violent video games to children without parental supervision (Brown v. EMA, 2011). The American Psychological Association (APA, 2020) also cited the mixed results in the field, and the resulting inability to make stable conclusions. With the research presented here, we aim to add new findings to the debate. On the strength of Ferguson and Wang (2019) and Devilly et al. (2021), we considered both personality traits linked to aggression and the characteristic of video exposure.

The first public debate over violence in video games began in the United States in the early-1990s. In 1993 and 1994, the United States Congress held two successive hearings on violence in video games (Walsh, 1993; S. Rep. No. 104-27, 1995; Blackburn, 2011). These hearings were sparked by parental concern regarding flagship games that exploited new and powerful technology. Two titles cited for having sparked this concern were Night Trap for the Sega CD, which used full-motion video to present violence against women, and Mortal Kombat, which used realistic digitised actor sprites and adjustable blood content. Video game companies, like SEGA, foresaw potential issues with players accessing content unsuitable for their age and introduced their own age rating system to counteract this issue; however, the rating system was considered too vague to be industry-applicable (Caron and Cohen, 2013). Following the 1994 hearing, the Entertainment Software Rating Board (ESRB) was established. The board’s primary goal was to rate video games on their content and age-appropriateness to safeguard children against violent and sexual content. Other regions followed suit, with the Pan European Game Information (PEGI) and Computer Entertainment Rating Organisation (CERO) (Computer Entertainment Rating Organisation, 2002) organisations created in Europe and Japan, respectively. Since then, younger players cannot purchase video games from retailers if they do not meet the age rating criterion, where rating systems are legally enforceable.

There have been considerable advances in the complexity, detail and distribution formats of violent video games since these regulations were introduced in the 1990s. In 2018, digital video game sales accounted for 83% of all video game sales (Clement, 2021). Digital content delivery makes it easier for players to buy games from the comfort of their homes, where it might be easier for a younger audience to access games developed for an older audience. For example, Steam, a computer storefront, asks for a simple confirmation of the date of birth without an identification check (Solorzano, 2018), whilst the Nintendo eShop for the Nintendo Switch does not check the player’s date of birth at all. Instead, the Nintendo Switch Parental Controls App lets parents decide what age ratings the child can access. However, only 39% of parents report using parental controls (Anderson, 2016). Thus, it appears that the regulatory system has not kept pace with the games’ use and distribution changes, giving rise to the question of whether it is still fit for purpose.

In short, the video games and aggression debate is controversial. New technologies have changed the level of immersion in video games and dramatically shifted the manner in which most users access the content. Yet, regulation appears not to have kept pace with this change. This study attempts to reintroduce the research question of whether violent video games cause aggression by considering personality variables previously unaccounted for, that are already considered in relation to aggression, such as narcissism and self-esteem, in the context of age-appropriate video game play.

Narcissism is characterised by excessive self-focus and self-interest. Narcissistic individuals are more likely to disregard others’ feelings to focus on themselves. Although research has shown that narcissism is linked to aggression, there is a disagreement as to whether aggression occurs more widely or is specific to the narcissism type. Researchers like Miller et al. (2021) suggest that most narcissists should be recognised as aggressive, but recent findings found a unique link between grandiose narcissism and aggression. This might not be the case, however, as Du et al. (2021) found that the relationship between narcissism and aggression differed depending on the level of both variables.

On the contrary, Kjærvik and Bushman (2021) found that all dimensions of narcissism (grandiose, vulnerable and entitlement) were related to aggression. Moreover, they found that this pattern of results occurred across many types of samples. They suggested that provocation might be a key factor in the relationship between narcissism and aggression. Although this interpretation disagreed with past findings, for example, Reidy et al. (2010), who found that individuals with high narcissism scores were more likely to be aggressors, further reviews, such as Lambe et al. (2016), agreed with this stance. They found that in a student sample that the link between narcissism and aggression was associated with an ego threat. This suggests narcissists must be provoked – perhaps by having their ego threatened – to evoke aggressive behaviours in them. Although independent studies provide mixed results, and reviews seem to disagree with each other, there is an overarching pattern of results: narcissism is somewhat related to aggression.

Although the literature on the relationship between narcissism and aggression is broad, not many studies have considered this in relation to video games, or violent video games. Blinkhorn et al. (2021) investigated the influence of their experimental game on the narcissistic perception of social exclusion. They found that the explosiveness feature of narcissism was correlated with a higher acceptance of violence in the context of social exclusion. This interpretation agreed with modern reviews of narcissism and aggression (Lambe et al., 2016; Kjærvik and Bushman, 2021) on the basis that narcissists must be provoked to evoke aggression-related behaviours and elaborated on their findings by suggesting that it is specifically ostracising social cues that were responsible for aggression in narcissists. However, little is known whether the extent of violence in video games influences this relationship. According to a conference paper by Melzer (2019), violence in video games did not meet the needs of individuals showing narcissistic attributes of the Dark Triad. However, no other investigations have been undertaken on the topic to date.

Self-esteem is a person’s positive or negative attitude towards oneself (Rosenberg, 1965a). Brummelman et al. (2016) suggested that narcissism and self-esteem were two distinct entities, only weakly correlated. Intuitively, they also suggested that narcissists saw themselves as superior but were unhappy with themselves, which might suggest low self-esteem. Therefore, our study will consider self-esteem as a variable separate from narcissism.

In the literature, there appears to be a lack of consensus amongst researchers studying the relationship between self-esteem and aggression, with modern studies distinguishing between different self-esteem types and obtaining significant results for the relationship between the two. Older studies, such as that of Bushman et al. (2009), showed that low self-esteem did not cause aggression, directly or indirectly. By contrast, when Descartes et al. (2019) administered self-esteem questionnaires to children and adolescents, they found that self-esteem was inversely related to aggression. However, Snowden et al. (2021) outlined the need to distinguish between types of self-esteem as the flagship instrument to measure self-esteem, the Rosenberg Self-esteem Questionnaire, which measured global self-esteem only. This sparked an investigation of the distinct effects of subcategories of self-esteem: agency and communion. Snowden et al. (2021) argued that different types of self-esteem displayed different associations with aggression. Namely, communion was negatively associated with aggression, whilst agency was related to aggression (but not reactive aggression, which is displayed in relation to threat). Further research by Amad et al. (2020) showed that low self-esteem was associated with reactive aggression.

There is also a lack of studies on the link between self-esteem, aggression and violent video games. As such, we broaden this literature review also to consider studies looking at the connection between general video game use, aggression and self-esteem. The most relevant study on this topic, Fling et al. (1992), is now three decades old. They found that the amount of video gameplay correlated with aggression but not self-esteem. However, some more recent studies have linked video games and self-esteem. For example, Cudo et al. (2019) investigated the predictors of problematic video gaming. They found that personal distress via the mediator of self-esteem was a significant predictor of problematic video gaming. This suggests that players who suffer from personal distress might engage with problematic video gaming more as a function of their self-esteem. This pattern of results could be accredited to escapism, defined as seeking refuge from reality through entertainment. Laconi et al. (2017) found that problematic players had higher scores of escape motivations and argued that playing video games might be a valid coping mechanism. It is not yet clear, however, whether video games are a coping mechanism for players with healthy levels of engagement with video games. Including self-esteem in investigating the link between violent video games and aggression might provide valuable insight into whether violent video games are a means of escapism in the wider population of gamers.

Our study also considers gaming motivation, as it might provide valuable insight into the relationship between video games and aggression. In broad terms, occasional players seem to be driven by extrinsic motivations (e.g., completing the game), whilst more dedicated players are driven by intrinsic motivations (e.g., satisfaction and enjoyment) (Reid, 2012). A commonality for both groups is deriving challenges from gaming (Reid, 2012; Kneer et al., 2018). Deeper investigations into dedicated players revealed a vast plethora of motivations. There are positive motivations for gameplay, such as socialisation or increased agency (Fuster et al., 2013; Kneer et al., 2018). However, there are also negative motivations for gameplay, such as griefing (causing inconvenience to another player) or virtual aggression (Kneer et al., 2018). In the space of video game addiction, the motivations for gameplay seem to be positive, for example socialisation and immersion (Zanetta Dauriat et al., 2011). However, some addicted players cite escapism as their motivation for gameplay (King and Delfabbro, 2009; Zanetta Dauriat et al., 2011). Belonging to a negative player class, such as an aggressive player, seem to put players at risk of developing a video game addiction (Hussain et al., 2015). It appears that virtual aggression is one of the motivations for video gameplay, but it is considered as a class of aggressive players, rather than aggressive motivation, that puts players at risk of addiction. As such, we believe the relationship between aggressive motivations within the video game, aggression and violent video games should be studied further.

Based on the intrinsic motivations for video gameplay, Teoh et al. (2020) have proposed a gaming motivation questionnaire, surveying video game players on their motivation for playing specific video game genres, delivering promising results for using this scale to study gaming motivations. We posit, however, that it might be more accurate to look at specific video game titles, rather than genres. Video games evolved so much that they began transcending genres. For example, Minecraft can fit into genres of action, adventure, sandbox and survival. We will therefore use this proposed questionnaire to investigate gaming motivations, modifying it to ask participants about specific video game titles, rather than genres. This will allow us to better understand the relationship between video games and aggression.

Taken together, there is a noticeable literature gap in the study of violent video games and aggression in relation to the personality variables of narcissism and self-esteem. Narcissism literature, although broader in this field, has failed to answer the question of whether the relationship between narcissism and aggression is stronger in violent video game players. The self-esteem literature, on the other hand, has ignored the link between self-esteem and aggression in the context of video games, even though there is now a march towards the belief that self-esteem and aggression are somewhat linked. Therefore, it is paramount to investigate whether both narcissism and self-esteem and aggression could be mediated by the extent of engagement with violent video games.

Violent video games often involve a multiplayer aspect, replicating a social environment virtually. It is therefore not implausible to suggest that narcissists who are socially excluded (either in real life due to their self-centeredness or interest in video games, or virtually when playing competitively) will display heightened aggression, as per the findings of Kjærvik and Bushman (2021) and Lambe et al. (2016). Similarly, individuals with low self-esteem could pick up violent video games as a coping mechanism to relieve their aggression toward virtual entities, as per the findings of Laconi et al. (2017).

**Figure 1:**
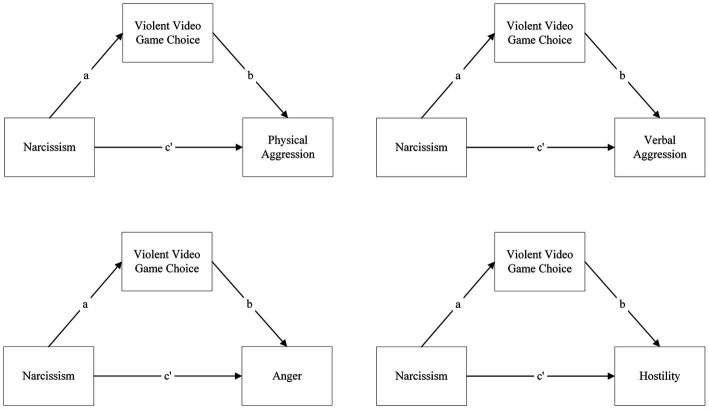
Models of simple mediations of violent video game choice on the relationship between narcissism and aggression dimensions: **(A)** physical aggression, **(B)** verbal aggression, **(C)** anger, and **(D)** hostility.

**Figure 2:**
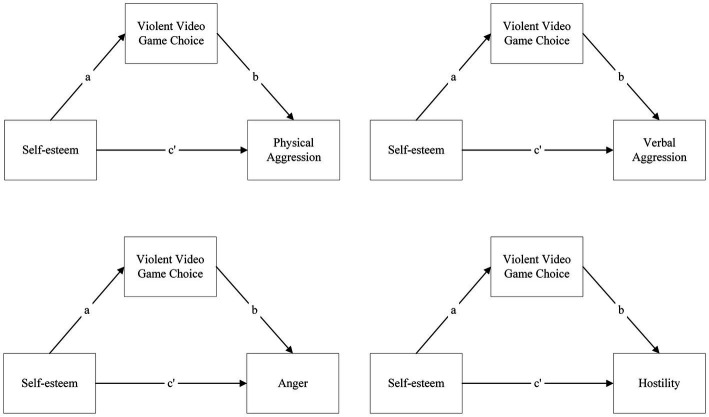
Models of simple mediations of violent video game choice on the relationship between self-esteem and aggression dimensions: **(A)** physical aggression, **(B)** verbal aggression, **(C)** anger, and **(D)** hostility.

As such, the primary aim is to investigate whether the relationship between narcissism and aggression dimensions or self-esteem and aggression dimensions is enabled solely by violent video game choice when it is considered a mediator (Figures 1, 2).

*H1a*: Violent video game choice will mediate the relationship between narcissism and aggression dimensions.

*H1b*: Violent video game choice will mediate the relationship between self-esteem and aggression.

**Figure 3:**
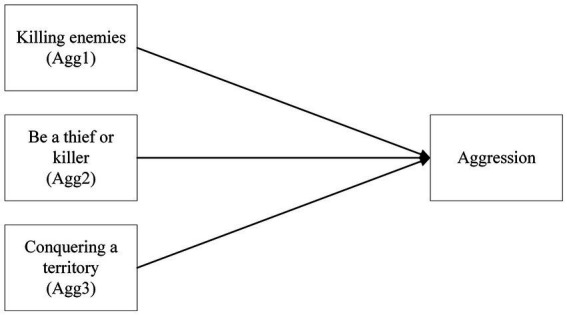
A model of the categorical regression of aggressive gaming motivations on aggression as a composite.

**Figure 4:**
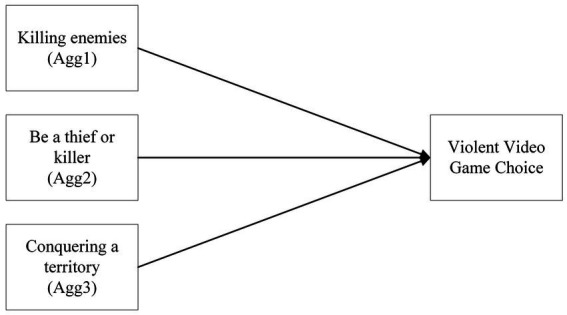
A model of the categorical regression of aggressive gaming motivations on violent video game choice.

The secondary aim is to investigate whether there will be a relationship between the aggressive gaming motivations, aggression, and violent video game choice. Aggression will be considered as a composite due to the nature of the surveyed aggressive gaming motivations concerning factors within the game (Figures 3, 4).

*H2a*: There will be a relationship between aggressive gaming motivations and aggression.

*H2b*: There will be a relationship between aggressive gaming motivations and violent video game choice.

## 2. Materials and methods

### 2.1. Participants

One hundred and sixty-six participants took part in the study, 113 males and 53 females, with age statistics of *M* = 25.2, *SD* = 8.05. The average amount of time spent playing video games per week was coded in increments of 1, where 0–5 h was assigned a value of 0, and 41+ hours was assigned a value of 8. The coded data has descriptive statistics of *M* = 3.65, *SD* = 2.07, which suggests that the average time spent playing games was around 20 h per week.

Participants volunteered to take part in the study after seeing an advert on Reddit or Discord. The study was posted on the Reddit communities r/samplesize, r/narcissism and r/truegaming; and on the Discord servers Cluster B Circus, r/NPD Official and NPD Recovery 2.0. Permission to post the link to the study experiment was granted by community moderators. The inclusion criterion was that only those who actively play video games should participate.

**Figure 5:**
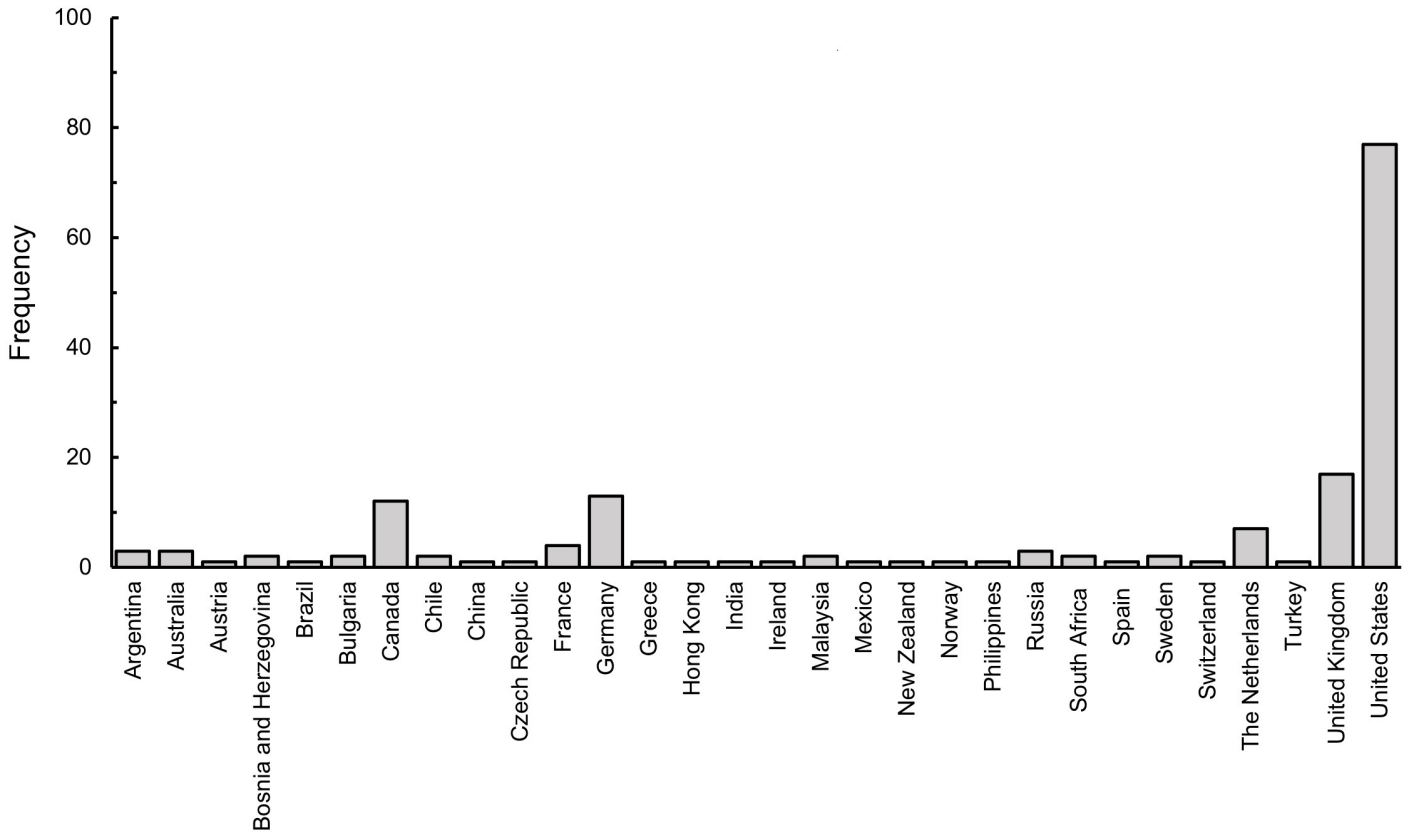
A frequency histogram of country of origin in the obtained sample.

The study was conducted in December 2021, as many countries, and especially those providing the biggest sample, had lifted their COVID-19 lockdown restrictions for the holiday season. Therefore, this study instigates the impact of video games post-lockdown. As seen in Figure 5, the United States was the most frequent country of origin in our sample (77 participants, 46.3% of sample). This was followed by the United Kingdom (34 participants, 20.4%), Canada (24 participants, 14.4%) and (22 participants, 13.2%) amongst the biggest countries in our sample. We can therefore assume that most of the sample came from Western cultures, and that at least some of the sample came from the region where PEGI is used (Figure 5).

### 2.2. Materials

The experiment was carried out on participants’ computers, using an online link to a Gorilla Experiment Builder questionnaire.

#### 2.2.1. Demographics questionnaire

This questionnaire collected data on age, gender, country of origin, three most played video games, and time spent playing video games per week.

#### 2.2.2. The Buss-Perry aggression questionnaire (BPAQ)

BPAQ was used to measure the level of aggression in each participant. This questionnaire was chosen as it is the preferred method of investigating aggression levels in psychological research (Buss and Perry, 1992).

The scale comprised 29 items on four dimensions, physical aggression (9 items, range 9–45), verbal aggression (5 items, range 5–25), anger (7 items, range 7–35) and hostility (8 items, range 8–40). Responses were collected on a Likert scale, from 1 (extremely uncharacteristic of me) to 5 (extremely characteristic of me). The composite range of this scale was 29–145. Two items (one from physical aggression and one from anger) were reverse scored. For H1a and H1b, this questionnaire will be analysed by splitting it into its dimensions. For H2a and H2b, this questionnaire will be analysed as a composite of all the dimensions. The internal validity of the BPAQ as a composite, measured by Cronbach’s alpha, was excellent, α = 0.901. The internal validity of the individual dimensions ranged between good and acceptable – good for anger, α = 0.819, good for physical aggression, α = 0.852, acceptable for hostility, α = 0.735, and acceptable for verbal aggression, α = 0.767.

#### 2.2.3. Narcissistic personality inventory-16 (NPI-16)

NPI-16 was used to measure narcissism levels in each participant. This scale estimates both healthy and unhealthy narcissism, where higher scores lean toward pathological narcissism. NPI-16 was preferred over other narcissism questionnaires due to its good consistency scores relative to its short form. It also enabled us to conduct a quick test of the level of narcissism that would not exhaust the attention span of the volunteers (Ames et al., 2006).

The scale comprised 16 statement pairs, and the participant chose whichever statement reflected their thoughts. The range of this scale was 0–1. The internal validity of the NPI-16, measured by Cronbach’s alpha, was acceptable, *α* = 0.768.

#### 2.2.4. The Rosenberg self-esteem scale (RSES)

RSES was used to measure the self-esteem of each participant. This questionnaire was chosen as it is the preferred and the most standardised self-esteem measure in psychological research. This questionnaire allows us to examine the escapism theory, as argued by Laconi et al. (2017).

The scale comprised 10 items. Responses were collected on a Likert scale, from 0 (strongly disagree) to 3 (strongly agree). The range of this scale was 0–30. The internal validity of the RSES, measured by Cronbach’s alpha, was excellent, *α* = 0.900 (Rosenberg, 1965b).

#### 2.2.5. The gaming instinctual motivational scale (GIMS)

GIMS was used to test for motivations behind video game engagement. Teoh et al. (2020) administered this questionnaire several times, asking for motivations based on video game genre. In our study, the scale was administered only once, and, in addition, participants were asked to provide motivation ratings for the game title of their preference.

The scale comprised 31 items split into 11 motivation types: survival, self-identification, collecting, greed, protection, aggressiveness, revenge, competition, communication, curiosity and colour appreciation. The responses were collected on a 5-point Likert scale, from 1 (never) to 5 (always). However, only the aggressiveness items were used in analyses. These were: “Killing enemies” (Agg1), “Be a thief or killer” (Agg2) and “Conquering a territory” (Agg3). These were analysed as ordinal variables, with range of 1–5. The internal validity of the GIMS as a composite, measured by Cronbach’s alpha, was excellent, α = 0.907. However, the internal validity of the aggressiveness dimension of GIMS was unacceptable, α = 0.316, and is likely a result of analysing single items, rather than the scale as a whole.

#### 2.2.6. Violent video game choice

We introduced a novel measure of violent video game choice. The researchers categorised the three most played video game titles provided by the participants as violent or non-violent based on the game’s PEGI rating. PEGI is currently the video game rating system used in Europe, established after the ESRB. It replaced many national rating boards to provide a unitary rating system across all European Countries, including the UK, and it is legally binding. PEGI ratings were sourced from the online PEGI database (Pan European Game Information, 2022). PEGI ratings span from PEGI 3 (suitable for all age groups) to PEGI 18 (suitable only for adults) (Pan European Game Information, 2017). PEGI uses content descriptors similar to the ESRB system used in the US.

As only 36.5% of video games bearing the ‘Violence’ content descriptor have been rated as suitable for more mature audiences (16+) (Pan European Game Information, 2022), the ‘Violence’ content descriptor was deemed inappropriate. Instead, the PEGI age ratings were used, as the descriptions of PEGI ratings include specific information on the type of violence that can be found within a given age rating scale. PEGI 12 was considered as the cut-off point for reasonable violence, as games in this category portray violence toward non-human characters or non-realistic violence toward human-like characters. PEGI 16 rating, instead, is given to games that portray violence similarly to how it would look in real life (Pan European Game Information, 2017). Thus, games with PEGI 3, 7 and 12 were classified as non-violent, and games with PEGI 16 and 18 were classified as violent.

The ESRB ratings were also collected as a secondary measure of violence. Certain games, usually games not released in the European market, or mobile games, lack PEGI ratings but have ESRB ratings. In such instances, we classified games as violent if they both had the ESRB rating of M 17+ and had the content descriptors of Violence or Intense Violence. ESRB ratings were sourced from the online ESRB database (Entertainment Software Rating Board, 2022).

Finally, if both the PEGI and ESRB ratings were not available, the researchers screened the game for its gameplay and assessed the gameplay against PEGI/ESRB ratings and descriptors, assigning them with a probable violent/non-violent judgement.

Participants reported three of their favourite video games. These would then be assessed against the PEGI and ESRB databases. This provided three dichotomous variables, where a value of 0 was given for non-violent games (≤PEGI 12) or 1 for violent games (≥PEGI 16). The three scores would then be summed up to produce a violent video game choice score in the range from 0 (plays 0 violent video games) to 3 (plays 3 violent video games).

### 2.3. Procedure

Participants volunteered to take part in the study after viewing a post on Reddit or Discord. They clicked on a link that sent them to the Gorilla experiment. They first read the Participant Information Sheet and provided informed consent. After, they were shown a screening question asking them whether they played video games. Participants were automatically rejected if they answered ‘No’ to this question. If they answered “Yes,” they would proceed to the demographic questionnaire. Upon completion, the participants would enter the testing phase. It comprised four questionnaires administered in a counterbalanced order using Gorilla’s Latin square Order node. These were: BPAQ, RSES, NPI-16 and GIMS. All questionnaires had to be completed to finish the testing phase. Participants had to click ‘Next’ to fully submit the data, after which the experiment was over.

### 2.4. Data analysis

Prior to data collection, the minimum sample size was calculated using G*Power (Faul et al., 2007). This was calculated for all the hypotheses to ensure the sample size was big enough to meet the statistical power assumptions of all tests. For all tests, the effect size was set at medium benchmark, *f*^2^ = 0.15, the error probability was set at α = 0.05 and the expected power was set at *power* = 0.80. For H1a and H1b, for both the a-path (1 tested predictor) and the b-path (2 tested predictors), the minimum sample size was 55. For H2a and H2b, for both the categorical regressions (3 predictors), the minimum sample size was 77. As such we have exceeded the minimum sample size requirements set out by *a priori* analyses, by recruiting 166 participants in total.

Participants who failed to complete all questionnaires were excluded from the dataset. The data was analysed with IBM SPSS 27. All mediation analyses were carried out using the PROCESS Macro package (Hayes, 2022) and checked for reliability with the standard SPSS Linear Regression tests using the method outlined in Speekenbrink (2021). We adopted the recommendation made by Baron and Kenny (1986): should any step of a mediation analysis return not significant results, the analysis will be stopped. All other analyses were carried out with the default SPSS 27 package.

Some data cleaning was performed. Firstly, the dataset was checked for outliers; none were flagged by the SPSS 27 package. Secondly, as noted earlier, some games given by participants (usually the case for mobile games) lacked PEGI ratings. A total of 57 data points out of 498 (11.4%) did not have associated PEGI ratings and were classified using the ESRB or direct observation of the gameplay. Thirdly, some participants reported playing fewer than three games. In such instances, the violent video game choice would be computed as though the missing games were non-violent (i.e., the missing values were assigned a value of 0). This method was employed as it was judged to be the most conservative.

## 3. Results

### 3.1. Descriptive statistics

As seen in Table 1, Anger and Physical Aggression were positively skewed, while Hostility and Verbal Aggression were normally distributed. Thus, the participants only demonstrated mild physical aggression and moderate anger. The narcissism scores were positively skewed and showed that most of the sample scored low on the narcissism scale, and therefore most of the sample displayed a ‘healthy’ level of narcissism. Self-esteem and Violent Video Game Choice (VVGC) were normally distributed. Players indicated playing 1.33 violent games if given an opportunity to report three of their favourite games.

**Table 1:** Descriptive statistics of variables in the present study.

Variables	*M*	SD	Skewness		Kurtosis	
			Statistic	SE	Statistic	SE
Narcissism	0.208	0.190	1.393	0.188	2.209	0.375
Self-esteem	15.963	5.958	0.129	0.188	−0.206	0.375
VVGC	1.331	1.005	0.309	0.188	−0.956	0.375
Anger	15.813	5.813	0.591	0.188	−0.207	0.375
Hostility	22.427	6.110	−0.089	0.188	−0.426	0.375
Physical Aggression	18.325	7.175	0.997	0.188	0.766	0.375
Verbal Aggression	15.301	4.552	−0.065	0.188	−0.658	0.375

### 3.2. Analyses

#### 3.2.1. Correlation analysis

Firstly, we carried out a correlation analysis to ensure all aggression dimensions were fit for further mediation analyses. As seen in Table 2, not all the dimensions of aggression were correlated with Narcissism and Self-esteem. For Narcissism, only Anger, Physical Aggression and Verbal Aggression returned significant correlations. For Self-esteem, only Hostility returned a significant correlation. As such, mediations will only consider the above variable pairings.

**Table 2:** A correlation matrix of variables of interest.

*r*	Narcissism	Self-esteem	VVGC	Anger	Hostility	Physical Aggression	Verbal Aggression
Narcissism							
Self-esteem	0.284**						
VVGC	0.023	0.003					
Anger	0.403**	−0.084	0.149				
Hostility	0.119	−0.516**	0.164*	0.491**			
Physical Aggression	0.267**	−0.039	0.131	0.615**	0.397**		
Verbal Aggression	0.487**	0.042	0.219**	0.527**	0.276**	0.391**	

* *p* < 0.05, ** *p* < 0.001.

Interestingly, VVGC returned a significant correlation for Hostility and Verbal Aggression. This would imply that violent video game choice influenced the hostility and verbal aggression scores.

#### 3.2.2. VVGC As a mediator between narcissism and anger

In Step 1 of the model, the direct effect of Narcissism on Anger, ignoring the mediator, returned a significant coefficient for Narcissism, *t*(164) = 5.64, *p* < 0.001, β = 0.403. However, in Step 2, the regression of Narcissism onto VVGC returned a not significant coefficient of Narcissism, *t*(164) = 0.299, *p* = 0.766, β = 0.123. Further steps were not analysed. There was no mediation of VVGC on the relationship between Narcissism and Anger.

**Figure 6:**
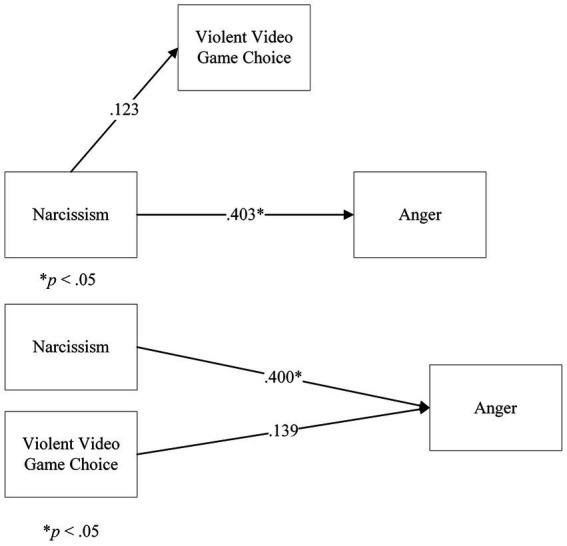
Analyses on Violent Video Game Choice, Narcissism, and Anger: **(A)** a mediation model of Violent Video Game Choice on Narcissism and Anger, **(B)** a multiple linear regression model of Narcissism and Violent Video Game Choice on Anger. Note: in the **(A)** panel, the b path is not shown, as the analysis was terminated.

We followed up this analysis with a multiple linear regression, considering Narcissism and VVGC as predictors of Anger. The overall model was significant, *F*(2,163) = 31.783, *p* < 0.001, *R*^2^ = 16.2%. The coefficient for Narcissism was significant, *β* = 0.400, *t*(164) = 5.63, *p* < 0.001. In contrast, the coefficient for VVGC was not significant, *β* = 0.139, *t*(164) = 1.97, *p* = 0.051. This showed that Narcissism predicted Anger. VVGC did not predict Anger (Figure 6).

#### 3.2.3. VVGC As a mediator between narcissism and physical aggression

In Step 1 of the model, the direct effect of Narcissism onto Physical Aggression, ignoring the mediator, returned a significant coefficient for Narcissism, *t*(164) = 3.53, *p* < 001, β = 0.267. However, in Step 2, the regression of Narcissism onto VVGC returned a not significant coefficient of Narcissism, *t*(164) = 0.299, *p* = 0.766, β = 0.123. Further steps were not analysed. There was no mediation of VVGC on the relationship between Narcissism and Physical Aggression.

**Figure 7:**
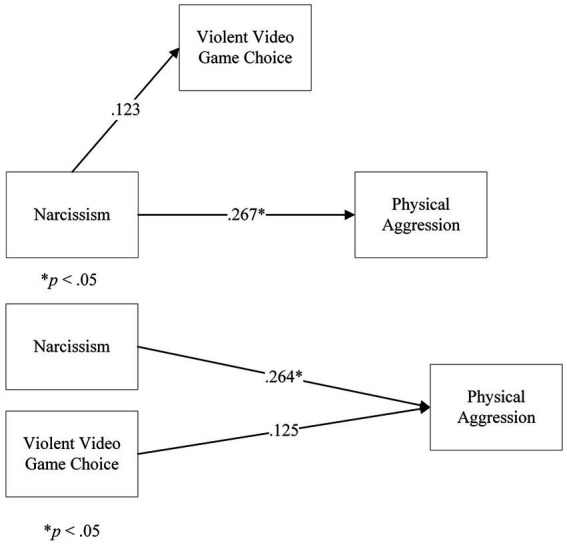
Analyses on Violent Video Game Choice, Narcissism and Physical Aggression: **(A)** a mediation model of Violent Video Game Choice on Narcissism and Physical Aggression, **(B)** a multiple linear regression model of Narcissism and Violent Video Game Choice on Physical Aggression. Note: in the **(A)** panel, the b path is not shown, as the analysis was terminated.

We followed up this analysis with a multiple linear regression, considering Narcissism and VVGC as predictors of Physical Aggression. The overall model was significant, *F*(2,163) = 7.76, *p* < 0.001, *R*^2^ = 7.6%. The coefficient for Narcissism was significant, β = 0.264, *t*(164) = 3.53, *p* < 0.001. In contrast, the coefficient for VVGC was not significant, β = 0.125, *t*(164) = 1.67, *p* = 0.097. This showed that Narcissism predicted Physical Aggression. VVGC did not predict Physical Aggression (Figure 7).

#### 3.2.4. VVGC As a mediator between narcissism and verbal aggression

In Step 1 of the model, direct effect of Narcissism on Verbal Aggression, ignoring the mediator, was significant, *t*(164) = 7.14, *p* < 0.001, β = 0.487. However, in Step 2, the regression of Narcissism onto VVGC returned a not significant coefficient of Narcissism, *t*(164) = 0.299, *p* = 0.766, β = 0.123. Further steps were not analysed. There was no mediation of VVGC on the relationship between Narcissism and Verbal Aggression.

**Figure 8:**
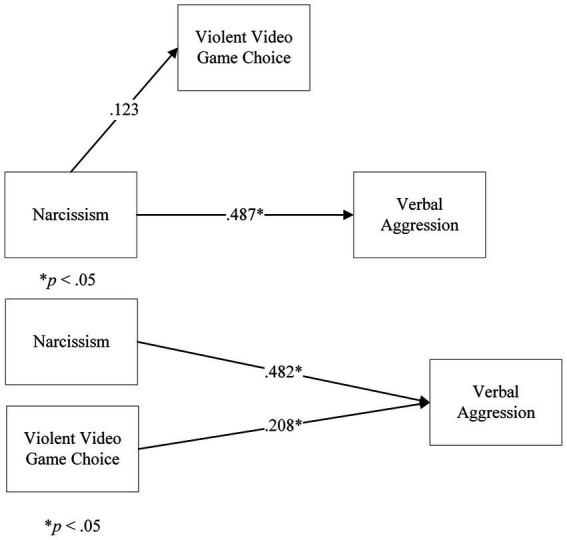
Analyses on Violent Video Game Choice, Narcissism and Verbal Aggression: **(A)** a mediation model of Violent Video Game Choice on Narcissism and Verbal Aggression, **(B)** a multiple linear regression model of Narcissism and Violent Video Game Choice on Anger. Note: in the **(A)** panel, the b path is not shown, as the analysis was terminated.

We followed up this analysis with a multiple linear regression, considering Narcissism and VVGC as predictors of Verbal Aggression. The overall model was significant, *F*(2,163) = 31.770, *p* < 0.001, *R*^2^ = 28.0%. The coefficient for Narcissism was significant, β = 0.482, *t*(164) = 7.257, *p* < 0.001. The coefficient for VVGC was also significant, β = 0.208, *t*(164) = 3.12, *p* = 0.002. This showed that both Narcissism and VVGC predicted Verbal Aggression (Figure 8).

#### 3.2.5. VVGC choice as a mediator between self-esteem and hostility

In Step 1 of the model, the direct effect of Self-esteem on Hostility, ignoring the mediator, was significant, *t*(164) = −7.84, *p* < 0.001, β = −0.20. However, in Step 2, the regression of Self-esteem onto VVGC returned a not significant coefficient for Self-esteem, *t*(164) = 0.039, *p* = 0.97, β = 0.001. Further steps were not analysed. There was no mediation of VVGC on the relationship between Self-esteem and Hostility.

**Figure 9:**
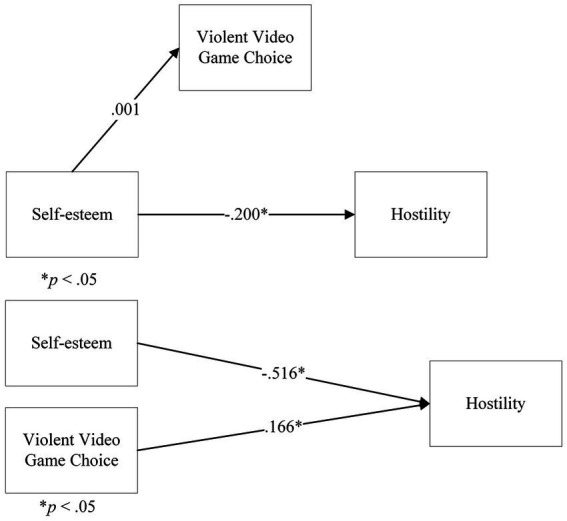
Analyses on Violent Video Game Choice, Self-esteem and Hostility: **(A)** a mediation model of Violent Video Game Choice on Self-esteem and Hostility, **(B)** a multiple linear regression model of Self-esteem and Violent Video Game Choice on Hostility. Note: in the **(A)** panel, the b path is not shown, as the analysis was terminated.

We followed this up with a multiple linear regression, considering Self-esteem and VVGC as predictors of Hostility. The overall model was significant, *F*(2,163) = 33.8, *p* < 0.001, *R*^2^ = 29.4%. The coefficient for Self-esteem was significant, β = −0.516, *t* = 7.84, *p* < 0.001. The coefficient for VVGC was also significant, β = 0.166, *t* = 2.52, *p* = 0.013. This showed that both Self-esteem and VVGC predicted Hostility (Figure 9).

#### 3.2.6. Relationship between aggressive gaming motivations and aggression as a composite

Categorical regressions were performed to investigate the relationship between aggressiveness motivations and aggression. Categorical regressions were suitable, as these convert ordinal variables (e.g., the GIMS motivations) into nominal variables that can be used to predict values of continuous variables (e.g., aggression scores/Violent Video Game Choice) via a linear regression.

**Figure 10:**
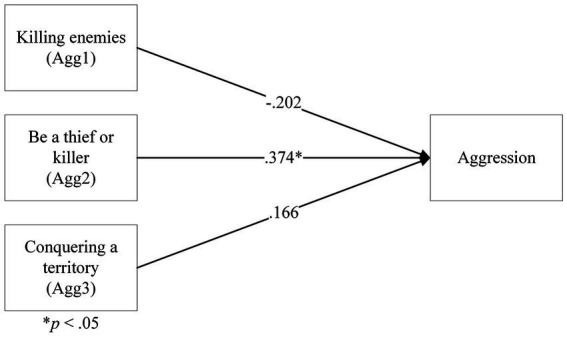
A categorical regression model of aggressiveness gaming motivations on composite aggression.

The categorical regression of aggressive motivations on aggression total score returned a significant model, *F*(7,158) = 6.37, *p* < 0.001, *R^2^* = 22%, However, upon the inspection of the coefficients, only Agg2 was significant, β = 0.374, *F*(3) = 19.2, *p* < 0.001. Agg1 and Agg3 were not significant, β = −0.202, *F*(1) = 2.16, *p* = 0.14, and β = 0.166, *F*(3) = 1.16, *p* = 0.33, respectively. This result suggested that only the ‘Be a thief or killer’ motivation was related to aggression (Figure 10).

#### 3.2.7. Relationship between aggressive gaming motivations and VVGC

As VVGC could include a value of 0, the categorical regression was set up with Impute missing values as Extra category to include values of 0 in the analysis.

**Figure 11:**
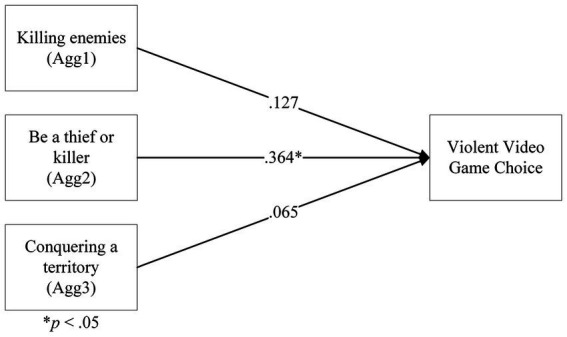
A categorical regression model of aggressiveness gaming motivations on violent video game choice.

The categorical regression of aggressive motivations on VVGC returned a significant model, *F*(6, 159) = 5.64, *p* < 0.001, *R^2^* = 14.4%. As with the regression on aggression, only Agg2 returned a significant coefficient, *F*(3) = 6.14, *p* < 0.001, β =0.364, and Agg1 and Agg3 were not significant, *F*(2) = 1.50, *p* = 0.23, β = 0.127, and *F*(1) = 0.241, *p* = 0.62, β =0.065, respectively. This result suggested that only the ‘be a thief or killer’ motivation predicted whether participants would be likely to play more violent video games (Figure 11).

## 4. Discussion

### 4.1. General discussion

Our study aimed to investigate whether video games classified as violent are related to aggression, considering personality traits often connected with aggression, such as self-esteem, narcissism, and gaming motivations.

**Figure 12:**
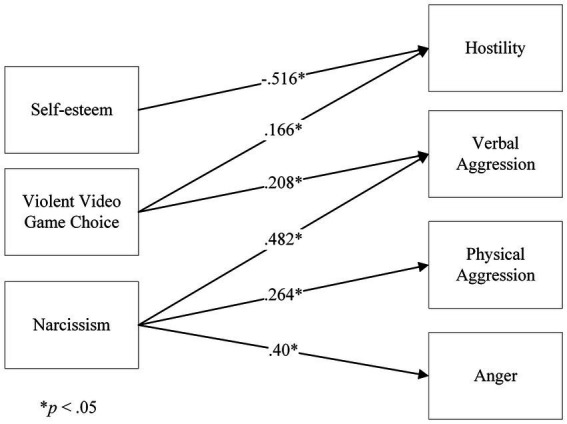
Overall results of significant analyses on the relationships between Self-esteem, Violent Video Game Choice, Narcissism and Aggression dimensions. The figure is not a structural equation model, but an aggregated result of all significant relationships for the variables of interest.

We found that violent video game choice was not a mediator of the relationships between narcissism/self-esteem and aggression components. However, we found that violent video game choice predicted hostility and verbal aggression, self-esteem predicted only hostility, while narcissism predicted hostility, physical aggression, and anger (see Figure 12).

These results show that in the gaming population, narcissism, self-esteem and violent video game choice, are predictors of different components of aggression. These results are novel and in line with the literature on narcissism (Kjærvik and Bushman, 2021) and self-esteem (Descartes et al., 2019), although they shed further light on the aggression components in the context of gameplay.

Interestingly, we obtained these results by classifying, both games with a rating of PEGI 16 (approaching real-life violence) as well as PEGI 18 (gross violence) as violent. This is a novel result that can inform future investigations into the relationship between video games and aggression. The findings contradict past literature. Cabras et al. (2019) found no evidence of the influence of violent video game choices on self-esteem and aggression when using the same measures. However, their sample comprised solely of Italian participants, while our study considered an international sample, mostly Western. In addition, they only considered PEGI 18 as a classifier for violence, whilst our inclusion of PEGI 16 ratings yielded a significant relationship between violent video game choice and aggression. The inclusion of PEGI 16 was triggered by their suggestion that further research should consider including the PEGI 16 rating.

The analysis of aggressive motivations in terms of aggression returned significant results, but this was solely due to the ‘Be a thief or killer’ motivation. The same pattern of results emerged when the same regression analysis was carried out on violent video game choice. We can speculate that players engage with more violent video game content to immerse themselves in roles that are undesirable in society, and that they perhaps would never undertake in the real-world. Thus, the fun of playing video games could also be doing things which one would not normally do in the real-world.

### 4.2. Theoretical implications

Our study opens a wider discussion about the possible impact of violent video game content on aggressive behaviour.

Online age verification is still widely underutilised in online media distribution and consumption. Cinemas enforce age restrictions for films that are not age-appropriate for children, and sometimes such films can be seen by young people only with adult supervision. This is still largely unaccounted for in Internet media. In fact, it is only recently that there has been a legislative drive to require age verification to view websites containing pornographic content in the United Kigdom (McCallum, 2022).

Many digital content distribution platforms, for both films and games, rely on parental guidance and discount age verification via identification (players are asked to input their date of birth at most). This can enable younger players to purchase games that are not age appropriate.

Considering the findings, the authors suggest digital content delivery platforms to consider revising their age verification procedures to prevent younger players from being exposed to inappropriate game material.

### 4.3. Limitations and future research

This study has a few limitations. The sample was largely composed of players from the United States. Although the diversity of the remaining part of the sample was broad, it is possible that our findings might only be applicable to the players from the United States.

Only one of the aggressiveness gaming motivations provided by GIMS returned significant results when assessed against the aggression scores. This might suggest that the aggressive motivations studied by GIMS might not have been relevant to aggression in our study. If this is true, this limits the conclusions that can be made regarding players’ motivations or suggest that these motivations might not be relevant to studying aggression.

The measures of personality traits might not have been sensitive enough to detect the granularity needed to make viable comparisons. Looking at narcissism specifically, NPI-16 was chosen due to its short-form presentation. Additionally, RSES has been argued to only consider global self-esteem (Snowden et al., 2021), and as such, we could not have made distinctions between self-esteem domains. This questionnaire was chosen, as it was the flagship measurement instrument that was short enough to administer to volunteers, trading off granularity in self-esteem.

Finally, our study did not incorporate any qualitative/mixed research methods which would have allowed participants to explain their gaming motivations in more detail.

### 4.4. Conclusions and future research

This study investigated the link between personality traits (narcissism and self-esteem), and violent video game choice. The results show that participants scored higher on the aggression scale the more violent games they played. Playing violent video games predicted verbal aggression, alongside narcissism, and hostility, alongside self-esteem. Furthermore, we found that narcissism was a predictor of verbal aggression, physical aggression and anger, whilst self-esteem was a predictor of hostility. With further research, violent video game choice, assessed by the summation of PEGI ratings classified as violent, can become a factor in a toolbox of the psychology of aggression and violence.

Thus, the authors suggest that greater consideration should be given to the player’s age and their frequent access to games displaying violence. In PEGI 12, which we considered as the cut-off for the non-violent games classification in this study, violence is committed against non-human characters. Future research might consider further lowering the cut-off point. Future research could seek to apply the current procedure to younger samples (age 17 and below) to see whether the results and implications of the present research still stand.

Furthermore, it is possible to sample narcissism and self-esteem binomially and analyse them in a between-subjects manner to observe significant results for the relationship between such variables, violent video game choice and aggression. As such, future studies should strive to collect binomial samples to investigate the disparity between the two extremes of the investigated variables. Moreover, future research should confirm whether violent video game choice is a superior method of analysing aggression in relation to video games than time spent playing per week.

## Data availability statement

The raw data supporting the conclusions of this article will be made available by the authors, without undue reservation.

## Ethics statement

The studies involving human participants were reviewed and approved by University College London Department of Information Study Ethics Chair. The patients/participants provided their written informed consent to participate in this study.

## Author contributions

The wider research question of violent video games and aggression was brought up by SO, who also reviewed the literature and prepared the online experiment. The experimental design and variables were chosen by SO and DR, who both carried out the data analysis and critically revised the manuscript prior to publication. All authors contributed to the article and approved the submitted version.
